# Case Report: Combination of Omalizumab and Dupilumab for Recalcitrant Bullous Pemphigoid

**DOI:** 10.3389/fimmu.2020.611549

**Published:** 2021-01-29

**Authors:** S. Morteza Seyed Jafari, Laurence Feldmeyer, Simon Bossart, Dagmar Simon, Christoph Schlapbach, Luca Borradori

**Affiliations:** Department of Dermatology, Inselspital, Bern University Hospital, University of Bern, Bern, Switzerland

**Keywords:** bullous pemphigoid, dupilumab, omalizumab, recalcitrant, treatment

## Abstract

Bullous pemphigoid (BP) is a blistering autoimmune skin disease. Omalizumab, a monoclonal antibody directed to IgE, showed a beneficial effect in treatment of recalcitrant BP in case series. More recently, dupilumab, an interleukin (IL)-4-receptor alpha antagonist, also showed promising preliminary results. We describe a patient with refractory BP who showed a complete response to a combination therapy with omalizumab and dupilumab.

## Introduction

Bullous pemphigoid (BP) is the most frequent autoimmune subepidermal blistering dermatosis affecting predominantly the elderly (1, 2). Although various therapeutic options exist, management of BP can be challenging (1). Furthermore, distinct immunosuppressive therapies may now pose additional risks in terms of COVID-19. Presence of tissue-bound and circulating anti-BP180 and anti-BP230 IgE autoantibodies in BP patients and findings in mouse models of BP have provided evidence that IgE autoantibodies have a pathogenicity role in BPs (2–6). Furthermore, type 2 pro inflammatory cytokines, including IL-4 and IL-13 contribute to tissue inflammation and damage in BP (7). Therefore, omalizumab, as a humanized monoclonal antibody directed to IgE, and dupilumab, as an interleukin (IL)-4 receptor alpha antagonist, might have a beneficial effect in BP (2, 4, 5, 7–10). Here, we describe a patient with severe recalcitrant BP successfully treated with a combination therapy with omalizumab and dupilumab, as add-on therapy.

## Report of the Case

A 70-year-old male had a 2-year-history of recurrent severely itchy, eczematous skin lesions and skin blistering. The diagnosis of BP was made based on the presence of typical clinical features, consistent histopathological findings (subepidermal blistering with a dermal eosinophilic cell infiltrate), positive direct immunofluorescence assessments (presence of linear IgG deposition along the epidermal basement membrane zone, but no IgE deposits) and the positive values of the enzyme-linked immunosorbent assay (ELISA)-BP180 (43.1 U/ml; normal range < 9 U/ml) (MBL, Japan). There was a normal blood eosinophil count and the serum IgE-level were slightly increased (73 kU/L; normal range <70 kU/L).

**Figure 1:**
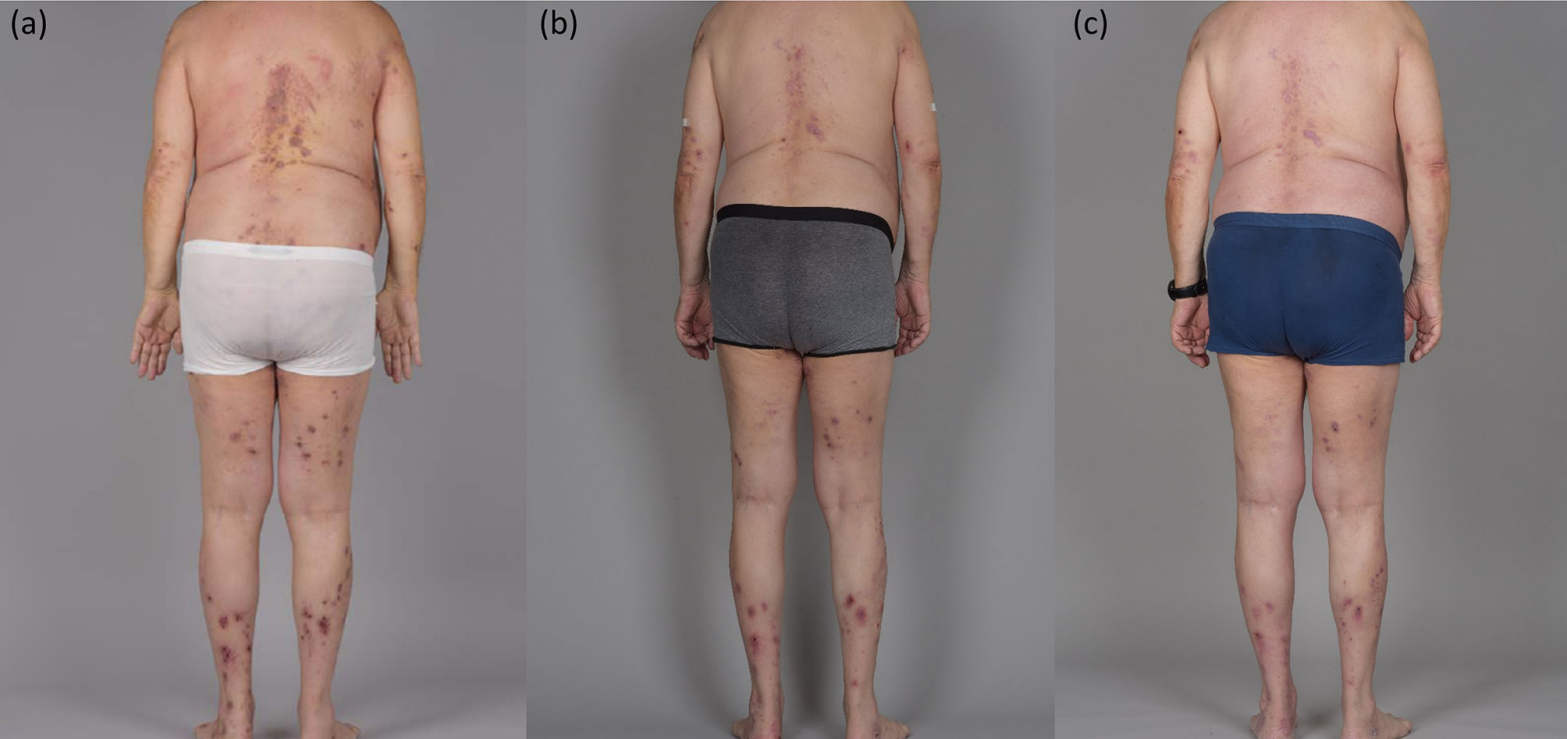
**(A)** The patient under mycophenolate-mofetil 2 g/day and topical corticosteroids (before therapy with dupilumab and omalizumab), **(B)** 2 months after addition of omalizumab therapy to his therapy protocol, **(C)** stable clinical results, 3 months after addition of dupilumab to his therapy protocol.

**Figure 2:**
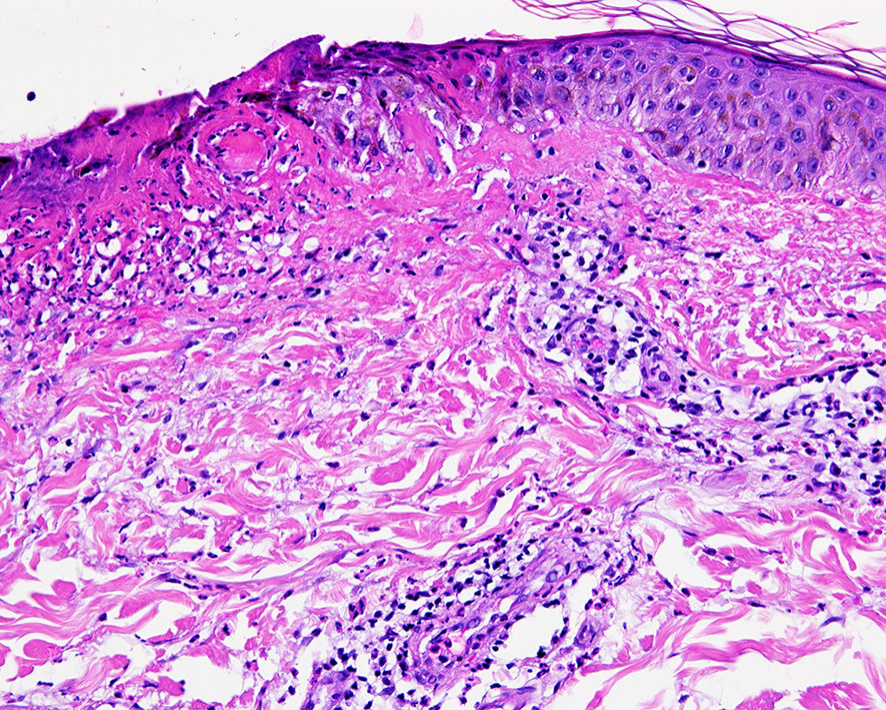
Histopathological image of the lesions, which is compatible with urticarial dermatitis with presence of ulceration and eosinophils.

The patient responded poorly to prior standard treatment regimens, including high potency topical corticosteroids and dapsone (up to 150 mg/day). Due to the patient’s uncontrolled metabolic syndrome (with obesity, type 2 diabetes mellitus, and arterial hypertension) and his age, the use of systemic oral corticosteroids was relatively contra-indicated. Furthermore, methotrexate therapy, 7.5 mg subcutaneously/weekly, had to be interrupted due to side effects after around 10 weeks. Mycophenolate-mofetil 2 g/day was subsequently administered during 9 months with only partial response of the disease and little impact on itch. Therefore, omalizumab (300 mg subcutaneously every 4 weeks) was added to his regimen of mycophenolate-mofetil and high potency topical corticosteroids. After two months of omalizumab, the pruritus Visual Analog Scale (VAS) improved from 9/10 to 2/10. The ELISA-BP180 values also significantly decreased (26.0 U/ml). However, the patient still suffered from mild itch with development of transient lesions and prurigo-like lesions. To achieve full disease control, the patient was also given as add-on therapy dupilumab, 600 mg subcutaneously initially followed by 300 mg SC every other week, while regimen of mycophenolate-mofetil, topical steroids and omalizumab remained unchanged. Under this novel combination therapy the pruritus disappeared (VAS 0/10) within three months, while no new inflammatory lesions developed. Complete healing of the excoriated lesions resulting in post-inflammatory dyschromia was observed. At the 7-month-follow up visit, the patient was in clinical remission, and mycophenolate-mofetil and topical corticosteroids were then stopped. Three months later, the patient remained in complete remission on combination of omalizumab and dupilumab with no clinical or biological side effects and ELISA-BP180 levels remained stable (24.8 U/ml). (**Figures 1**, **2**).

## Discussion

Management of BP can be challenging (2). Here, we present for the first time the concomitant use of dupilumab and omalizumab for a patient with severe treatment-resistant BP, which showed no improvement to high potency topical steroids, dapsone, and mycophenolate-mofetil. Some studies described that high serum levels of IgE correlate with disease severity in BP. However, in our patient there was no significantly increased serum IgE level. The observed beneficial effect of omalizumab in our patient with an excellent clinical response might be related to the sequestration of the free IgE, down-regulation of FcϵRI expression, dissociation of the IgE-FcϵRI complex and finally decrease in FcϵRI concentration on eosinophils and mast cells in affected tissues (6). Furthermore, the majority of patients with BP have an increased number of cells producing IL-4 and IL-13 in blood and lesional skin. The latter have been shown to decline with disease improvement (7, 11). Based on this knowledge, dupilumab thus provides an option to target these disease-driving mechanisms inhibiting IL-4 and IL-13 signaling. It also indirectly downregulates IgE secretion and eosinophil activity (7, 11).

Pruritus is also a common major complaint of BP patients. Dupilumab is thought to ameliorate pruritus by decreasing peripheral itch sensory neuron signals due to its direct effects on IL-4 and IL-13 and its effects on eosinophils resulting in decreased secretion of IL-31 (7, 10, 12). The efficacy of dupilumab in successfully controlling the pruritus highlight a specific clinically important Th-2 pathway, which drives pruritus both in atopic dermatitis and other inflammatory diseases, including BP (10).

Despite the obvious limitation of our observation, the combination of omalizumab and dupilumab might be extremely valuable in therapy-recalcitrant BP because of their good safety profile, which may be relevant during the during the COVID-19 pandemic. Controlled studies are needed to define the role of anti-IgE blockade and an interleukin-4 receptor antagonist either alone or in combination as add-on therapy in BP.

## Data Availability Statement

The original contributions presented in the study are included in the article/supplementary materials, further inquiries can be directed to the corresponding author.

## Ethics Statement

Ethical review and approval was not required for the study on human participants in accordance with the local legislation and institutional requirements. Written informed consent was obtained from the individual for the publication of the images or data included in this article.

## Author Contributions

SMSJ, LF, SB, DS, CS, and LB designed the study and performed the acquisition, analysis, and interpretation of data. SMSJ and LB wrote the manuscript. SMSJ, LF, SB, DS, CS, and LB performed critical revision of the manuscript for important intellectual content. All authors contributed to the article and approved the submitted version.

## Funding

This work was supported by the Department of Dermatology, Bern University, Bern, Switzerland.

## Conflict of Interest

The authors declare that the research was conducted in the absence of any commercial or financial relationships that could be construed as a potential conflict of interest.
